# The Cross-Education Phenomenon: Brain and Beyond

**DOI:** 10.3389/fphys.2017.00297

**Published:** 2017-05-10

**Authors:** Ashlee M. Hendy, Séverine Lamon

**Affiliations:** School of Exercise and Nutrition Sciences, Institute for Physical Activity and Nutrition, Deakin UniversityGeelong, VIC, Australia

**Keywords:** endocrine system, hypertrophy, neural plasticity, rehabilitation, resistance training, skeletal muscle

## Abstract

**Objectives:** Unilateral resistance training produces strength gains in the untrained homologous muscle group, an effect termed “cross-education.” The observed strength transfer has traditionally been considered a phenomenon of the nervous system, with few studies examining the contribution of factors beyond the brain and spinal cord. In this hypothesis and theory article, we aim to discuss further evidence for structural and functional adaptations occurring within the nervous, muscle, and endocrine systems in response to unilateral resistance training. The limitations of existing cross-education studies will be explored, and novel potential stakeholders that may contribute to the cross-education effect will be identified.

**Design:** Critical review of the literature.

**Method:** Search of online databases.

**Results:** Studies have provided evidence that functional reorganization of the motor cortex facilitates, at least in part, the effects of cross-education. Cross-activation of the “untrained” motor cortex, ipsilateral to the trained limb, plays an important role. While many studies report little or no gains in muscle mass in the untrained limb, most experimental designs have not allowed for sensitive or comprehensive investigation of structural changes in the muscle.

**Conclusions:** Increased neural drive originating from the “untrained” motor cortex contributes to the cross-education effect. Adaptive changes within the muscle fiber, as well as systemic and hormonal factors require further investigation. An increased understanding of the physiological mechanisms contributing to cross-education will enable to more effectively explore its effects and potential applications in rehabilitation of unilateral movement disorders or injury.

## Introduction and overview

Resistance training is a popular form of physical exercise that involves repetitive muscular contractions performed against an external load. Resistance training is widely utilized in clinical rehabilitation and community settings in order to increase muscular strength, size, and overall physical function. The increase in strength that is observed following resistance training programs is due to a combination of morphological adaptations and neurophysiological activation of the targeted muscle (Folland and Williams, 2007). In 1894, the Yale Physiological Laboratory first reported observations of strength gains in the untrained contralateral limb following unilateral resistance training, terming the phenomenon “cross-education” (Scripture et al., 1894). In more recent times, the investigation of the characteristics and mechanisms underpinning cross-education have received substantial interest from the scientific community. This is particularly due to the potential for the application of cross-education in rehabilitation following unilateral injury or immobilization (Farthing et al., 2009; Hendy et al., 2012; Magnus et al., 2014).

Despite strong evidence confirming the existence of cross-education (Munn et al., 2004, 2005), the mechanisms underlying strength gain in the untrained limb remain somewhat unresolved (Lee and Carroll, 2007; Ruddy and Carson, 2013). The role of the nervous system has long been acknowledged, with modern technology now allowing for direct measurement of neurophysiological adaptations. However, few studies have examined the role of peripheral muscle factors and the contribution of systemic responses such as the release of anabolic hormones and myokines. In this review, we aim to explore and discuss existing knowledge surrounding the proposed neurological, muscular, and systemic factors underpinning cross-education. This multidisciplinary approach will allow a better understanding of the physiological mechanisms responsible for the cross-education effect.

## Characteristics of cross-education

A myriad of studies describing cross-education in both upper and lower limbs following various types of resistance training interventions have been conducted, including heavy load voluntary contractions (Munn et al., 2005; Goodwill and Kidgell, 2012; Latella et al., 2012), ballistic motor tasks (Lee et al., 2010; Hinder et al., 2013), electrically stimulated contractions (Hortobágyi et al., 1999) and motor imagery (Yue and Cole, 1992). The magnitude of strength transfer differs between studies, which may primarily be due to differences in training variables such as exercise complexity, contraction type, volume, intensity, and duration. Characteristics of the target muscle, strength testing methodology, and training status of participants may also influence the degree of strength increase reported. One meta-analysis revealed that the typical magnitude of strength gain in the untrained limb is 7.6% (when compared to pre-training), equating to approximately 50% of strength gain observed in the trained limb (Munn et al., 2004; Carroll et al., 2006). Given the potential for cross-education to be applied in clinical rehabilitation settings, there is a need to further understand the effect of individual training parameters, such as frequency, intensity, contraction type, and velocity, in order to optimize strength transfer.

Several studies have reported that cross-education is maximized when eccentric training paradigms are employed (Hortobágyi et al., 1997; Lepley and Palmieri-Smith, 2014). It has been postulated that the increased discharge rate of sensory stretch receptors (Ia afferents) that occurs with eccentric contractions may be responsible for increased strength transfer (Hortobágyi et al., 1997). Interestingly, unilateral training performed during whole body vibration, which is also believed to increase Ia afferent feedback, did not enhance the magnitude of strength transfer (Goodwill and Kidgell, 2012). Unilateral eccentric contractions also produced greater changes in cortical excitability and inhibition (Howatson et al., 2011), suggesting that contraction-specific differences in the descending motor command, rather than afferent input, may be responsible for this observation. Collectively, neurological evidence to explain the difference in strength transfer based on contraction type are somewhat conflicting (Howatson et al., 2011). This warrants further investigation of other unique properties of eccentric contractions, such as the endocrine response and its effects on skeletal muscle fibers. It has also been noted that strength gains are greater when the muscle action (eccentric, concentric, or isometric) used in strength testing is specific to the muscle action performed in training (Hortobágyi et al., 1997; Lepley and Palmieri-Smith, 2014). This is in line with general principles of resistance training specificity, and should be considered not only in study design and methodology, but also when prescribing unilateral resistance training for functional rehabilitation purposes.

The use of externally-paced training with an audible metronome has produced large magnitudes of cross-education in the lower limb in healthy, untrained individuals (Goodwill and Kidgell, 2012). This may be due to the increased cognitive demand and control of movement pattern, which likely results in greater use-dependent neuroplasticity (Ackerley et al., 2011; Leung et al., 2015). For example, a recent study compared externally-paced vs. self-paced unilateral bicep curls, and found that only externally-paced contractions influenced corticomotor output of the “inactive,” ipsilateral motor cortex (iM1) (Leung et al., 2015). Interestingly, the increase in corticospinal excitability and reduction in intracortical inhibition, determined with transcranial magnetic stimulation (TMS), replicated responses that were induced by a skill-based visuomotor tracking task (Leung et al., 2015). At present, there is no direct comparison between the magnitude of cross-education occurring following externally-paced vs. self-paced contractions delivered in a volume controlled training program. Thus, the benefits of utilizing externally-paced training when applying cross-education for rehabilitation remain somewhat speculative.

## Neurological mechanisms for strength transfer

Two theoretical models involving neural plasticity in the cortical regions of the brain have been proposed to explain the cross-education phenomenon (see Ruddy and Carson, 2013 for detailed review). The “bilateral-access” hypothesis involves the development of motor engrams following unilateral movements, which can be accessed not only by the trained limb, but also by the untrained limb. This explanation is derived from the observation of cross-education following unilateral practice of skilled-based perceptuomotor tasks, which have been studied extensively from a motor learning perspective (often referred to as “bilateral transfer”). Since effective production of force (i.e., muscular strength) involves a process of motor learning relating to effective recruitment of motor units, including inhibition of antagonists and co-ordination of synergists (Lee and Carroll, 2007), this concept can be applied to cross-education of strength. In contrast, the “cross-activation” hypothesis is based on the concept of unilateral contractions being driven by bilateral cortical activity in both the contralateral motor cortex (cM1) and iM1, producing lasting neuroplasticity in both cortices. Both hypotheses, which may not be mutually exclusive, acknowledge the importance of the iM1 in mediating the cross-education of strength, with distinction between the two models likely to be task dependent (Lee and Carroll, 2007; Lee et al., 2010; Ruddy and Carson, 2013). Strength based tasks requiring maximal force and motor unit recruitment may be mediated by cross-activation, while the bilateral-access model may rather contribute to transfer in tasks requiring complex sequencing or sensorimotor integration (Lee et al., 2010; Ruddy and Carson, 2013).

Crucial to the cross-activation hypothesis are the early observations of a concept termed “motor irradiation.” This describes the spill-over of unintended motor activity to the resting limb during forceful unilateral contractions, sometimes resulting in mirror movements and increased background electromyography (EMG) (Carson, 2005). While motor irradiation is an obvious candidate for inducing training related adaptations in the untrained limb, activity in the resting muscle is much lower than what would typically be required to elicit strength gains. Indeed, cross-education can occur in the absence of mirror movements or any observable increase in EMG (Carson, 2005; Hortobágyi et al., 2011). Early TMS studies enabled a more direct line of investigation into the origin of motor irradiation, revealing an increase in corticospinal excitability of the iM1 during moderate to strong unilateral contractions (Stedman et al., 1998; Muellbacher et al., 2000). It is now generally accepted that cross-activation of the iM1, rather than the resulting motor irradiation, mediates cross-education.

The use of neuromodulatory techniques, such as repetitive TMS (rTMS) and transcranial direct current stimulation (tDCS), has allowed further probing of the role of both the iM1 and cM1 in cross-education. Both techniques involve the delivery of non-invasive, painless stimulation to the scalp, which influences the excitability of underlying neurons. In one study, low-frequency rTMS was utilized to downregulate the iM1 following unilateral ballistic muscle contractions. It was reported that both performance gains and changes in neural excitability of the untrained limb were abolished (Lee et al., 2010). Conversely, when the same stimulation was applied to the cM1, excitability and performance gains in the trained limb were reduced, while transfer to the untrained limb remained unaffected (Lee et al., 2010). It was concluded that the iM1, and not the cM1, was primarily responsible for the acute transfer effects observed following early practice of a novel ballistic motor task (Lee et al., 2010). In our laboratory, anodal tDCS has been applied in a manner that up-regulates the excitability of underlying neurons. In one study, stimulation was applied to the iM1 during a single bout of heavy load dynamic wrist extensions, resulting in increased corticomotor excitability, reduced intracortical inhibition and a modest but significant increase in 1RM strength of the untrained limb (Hendy and Kidgell, 2014). When the same stimulation protocol was applied during sessions across a 2-week unilateral training program for the biceps brachii, strength gains in the untrained limb, and the associated neural adaptations, exceeded that observed in a group receiving sham-stimulation (Hendy et al., 2015). Both approaches support the cross-activation hypothesis, and provide evidence that the iM1 plays an integral role in cross-education of strength.

Few studies have investigated the function of spinal reflexes in the untrained limb following unilateral strength training programs. The electrically evoked Hoffman reflex (H-reflex) can be used to assess excitability of the Ia afferent motor neuron pathway in the untrained limb. In studies where cross-education of strength was reported, the maximal amplitude of the H-reflex in the untrained agonist muscle was unchanged (Lagerquist et al., 2005; Fimland et al., 2009; Dragert and Zehr, 2011). In a detailed examination of H-reflex adaptations following unilateral training of the ankle dorsiflexors, authors reported a non-significant trend for increased H-reflex amplitude at threshold in the tibialis anterior (reflecting recruitment of low-threshold motor units), alongside a decrease in the maximal H-reflex amplitude of the untrained soleus (antagonist muscle). Authors concluded that cross-education was associated with subtle changes in spinal reflexes (Dragert and Zehr, 2011). The volitional wave (V-wave), which reflects efferent neural drive during muscular contraction, has been shown to increase in the untrained following unilateral training (Fimland et al., 2009). It is essential to recognize that while this observation indicated an increase in excitability of the spinal motor neuron pool, this difference is still likely to be a result of net increase in drive from the iM1. Collectively, results from these studies suggest that changes in spinal circuitry may only play a small role in the cross-education effect (Lagerquist et al., 2005; Fimland et al., 2009; Dragert and Zehr, 2011). Furthermore, it has been suggested that the functional requirement for reciprocal inhibition during gait may result in greater implications for the spinal reflex arc in the lower limb (Dragert and Zehr, 2011), with all studies to date examining the H-reflex in either soleus or tibialis anterior. The effects of single limb training on spinal reflexes in the upper limb is yet to be determined.

Recently, it has been hypothesized that the mirror-neuron system may play a role in cross-education (Howatson et al., 2013; Zult et al., 2014). Mirror neurons provide a neuroanatomical link between visual sensory inputs and motor neurons that produce descending drive during execution of movements, without explicitly resulting in movement imitation (see Zult et al., 2014 for review). To assess whether visual stimuli has the ability to augment cross-education, several studies have now investigated motor performance and neurophysiological responses to the unilateral contractions performed with a mirror box to simulate movement of the inactive limb (Garry et al., 2005; Reissig et al., 2014, 2015; Zult et al., 2015, 2016). One study employed a 3 week high intensity concentric training protocol in the wrist flexors, reporting that the magnitude of strength gain in the untrained limb was almost doubled when training was performed with a mirror when compared to training without visual stimuli (61 and 34% strength increase respectively; Zult et al., 2016). In contrast, 300 ballistic finger movements performed unilaterally during a single training session produced a similar improvement in contralateral motor performance and corticospinal excitability (measured with TMS), regardless of whether visual mirror feedback was provided (Reissig et al., 2015). While there is some evidence from TMS studies for increased corticospinal excitability of the motor pathway to the inactive limb during mirror illusions (Garry et al., 2005), more recent work suggests that a reduction in intracortical inhibition of the iM1 may be the primary factor underpinning any benefits associated with mirror training (Reissig et al., 2014; Zult et al., 2015). Overall, the hypothesis for involvement of the mirror neuron system in unilateral training supports the notion that increased neural output from the “untrained” motor cortex plays a key role in cross-education.

## Cross-transfer effects on skeletal muscle

Beyond the well-described effects of unilateral training on neurological plasticity, there is surprisingly little knowledge surrounding cross transfer effects in skeletal muscle. As is the nervous system, skeletal muscle is a plastic tissue able to rapidly modify its structure, function, and metabolism in response to internal and external stress signals. A large majority of cross-education studies have reported direct and indirect evidence for adaptations of the neural pathways innervating the untrained muscle, but few associations have been drawn between changes in strength and alterations in muscle structure and composition.

An early study demonstrated that in 14 elderly males undergoing a 12-week unilateral weight-lifting program, the mean cross-sectional area (CSA) of fast-twitch (type II) fibers increased in both the trained and untrained biceps brachii muscle, although to a greater extent in the trained muscle (Brown et al., 1990); a finding not replicated by others (Houston et al., 1983). However, this result was paralleled by an increase in the muscle diameter of the elbow flexors (biceps brachii and brachialis) in the trained, but not the untrained limb. In line with these early data, most studies have not observed any increase in muscle size in the untrained muscle or group of muscles as a result of cross-education in an immobilized (Farthing et al., 2011) or untrained (Bezerra et al., 2009; Magnus et al., 2014; Kurobe et al., 2015) opposite limb, leading to the continued perusal of neurological factors.

In contrast, a number of studies have described that unilateral training can prevent disuse-induced muscle atrophy in the untrained muscle. A study including 30 young (20–26) male and female participants showed that following 3 weeks of unilateral immobilization and maximal isometric ulnar deviation training of the opposite wrist 5 days per week, training the contralateral arm prevented the loss of muscle thickness of the immobilized posterior medial forearm (flexor carpi ulnaris and flexor digitorum superficialis; Farthing et al., 2009). Similarly, muscle thickness was assessed in 25 young (20–25) males and females completing a maximal isometric elbow flexion and extension training program 3 days per week during 4 weeks while their other arm was immobilized (Magnus et al., 2010). While biceps and triceps brachii thickness significantly increased in the trained arm when compared to the control, non-intervention group, a positive adaptation was also observed in the non-trained arm. This change could not be imputed to a net gain in muscle mass, as it was non-significantly different from the control group. However, the loss of muscle mass observed in the immobilization group that did not train was significantly prevented by contralateral training (Magnus et al., 2010). These findings were reproduced by a study from our group that investigated the effects of 3 weeks of unilateral arm curl strength training 3 days per week while the other arm was immobilized in 28 young participants. Immobilization alone resulted in a reduction in the thickness of the elbow flexors (biceps brachii and brachialis). This loss of muscle mass was counteracted when the contralateral arm was subjected to strength training (Pearce et al., 2013). Although these results are somewhat encouraging with respect to the potential benefits of cross-education to prevent muscle atrophy during immobilization, the limitations associated with the use of ultrasound for indirectly assessing muscle CSA (via muscle thickness) should be acknowledged. At present, no studies have utilized magnetic resonance imaging (MRI) or peripheral quantitative computed tomography (pQCT), considered “gold standard” measures, to detect changes in muscle mass during cross-education and immobilization.

One unique study investigated the collective gene expression pattern following a 1 h unilateral electro-stimulation intervention in rat soleus muscle (Pimenta et al., 2009). Electro-stimulation increased the expression of 26 genes in the stimulated muscle only, and 66 genes in both the stimulated and the contra-lateral muscle. The magnitude of these increases was generally smaller in the contra-lateral muscle. The overexpressed genes were typically stress-response genes, including members of the heat-shock family of proteins (Hsp), and metabolic genes. Several members of the MAPK (mitogen-activated protein kinases) family of proteins displayed an increase in mRNA in both muscles. The MAPK pathway is a well-described positive regulator of muscle mass (Widegren et al., 2001), suggesting that MAPK may provide a link between contraction-induced stimulation of the contra-lateral muscle and protection against muscle atrophy. Another hypothesis relies on the potential role of Hsp in compensatory muscle hypertrophy (Kawada and Ishii, 2005); both theories however warrant further experimental validation. Rabbits subjected to 6 weeks of unilateral electro-stimulation training reported minor changes in muscle fiber composition as well as in markers of muscle inflammation and muscle damage in both the stimulated and the non-stimulated leg. Bearing in mind the possible excessive duration and intensity of this protocol, these observations suggest that cross-transfer effects can also lead to deleterious muscle adaptions (Song et al., 2014).

## Possible mechanisms for positive adaptations of the contralateral muscle

**Figure 1:**
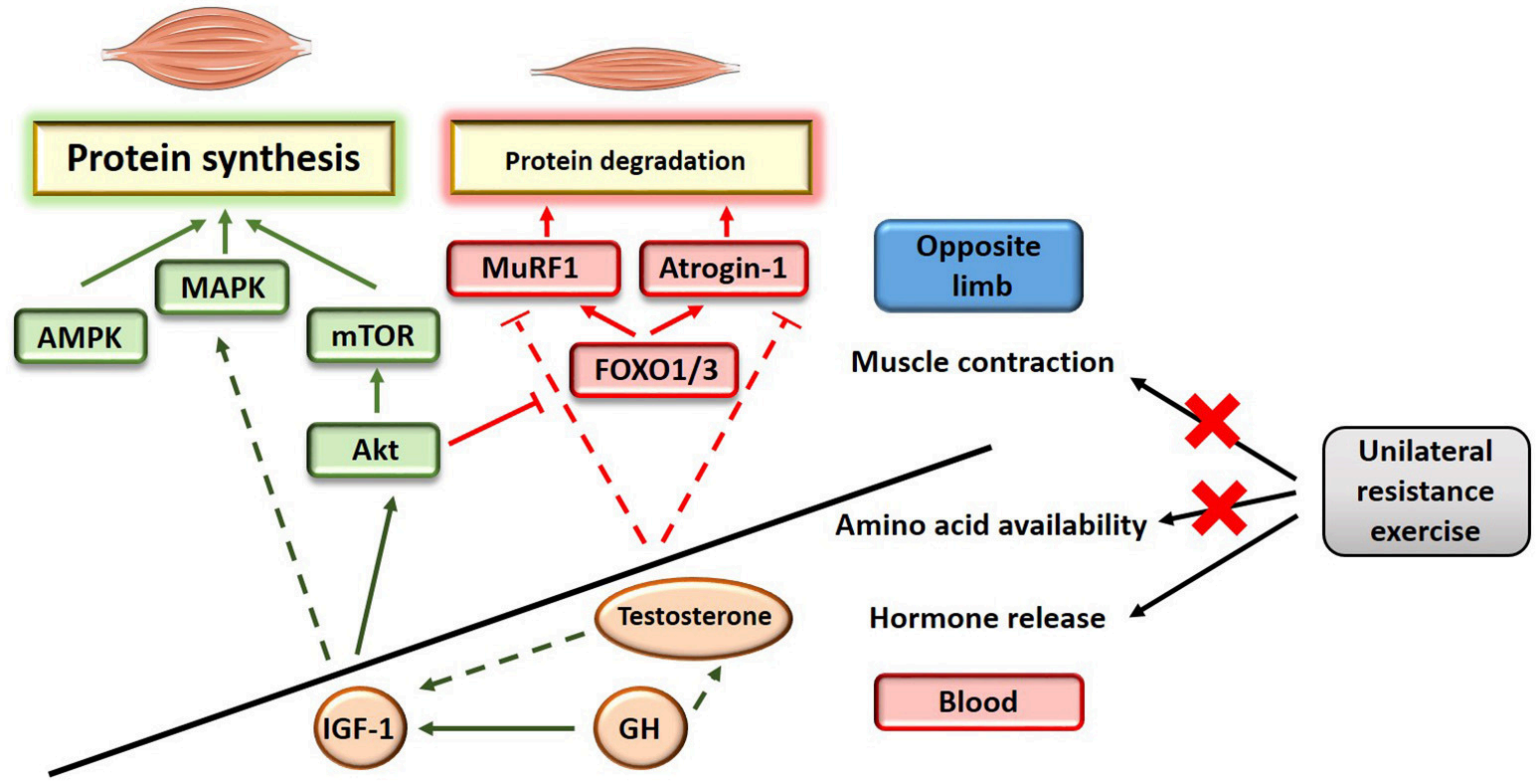
**Schematic illustration of the potential mechanisms influencing muscle protein balance as a result of unilateral training**. Full line indicates a direct effect; dashed line indicates an indirect effect.

The mechanisms underlying the attenuation of unloading-induced muscle atrophy as a result of cross-education have not been investigated. The maintenance of skeletal mass relies on the fine balance existing between muscle protein synthesis and muscle protein degradation (proteolysis) (Russell, 2010). Muscle atrophy is reflective of a disrupted equilibrium where more proteins are being degraded than being synthesized in the muscle. At the molecular level, muscle protein synthesis is synergistically regulated by the Akt/mechanistic target of rapamycin (mTOR) pathway (Schiaffino et al., 2013), the mitogen-activated protein kinases (MAPK) pathway (Widegren et al., 2001) and the Ca^+2^-dependant 5' AMP-activated protein kinase (AMPK) pathway (Schiaffino et al., 2013). Akt also inhibits muscle proteolysis by repressing the action of the forkhead box (FOXO) proteins and their targets, the muscle specific E3-ubiquitin ligases MAFbx (atrogin-1) and muscle RING-finger protein 1 (MuRF1) (Schiaffino et al., 2013; see Figure 1). These molecular signaling events are tightly controlled by a combination of anabolic and catabolic stimuli including (1) the presence or absence of amino acids, (2) the presence or absence of neuronal stimulation, inducing a change in intracellular Ca^+2^ concentration and determining muscle contraction, and (3) peripheral factors including metabolic hormones and myokines. It is expected that the protective action of cross-education on skeletal muscle mass relies on a combination of mechanisms that can synergistically activate the protein synthesis pathways and/or inhibit the protein degradation pathways. Why a positive outcome on muscle mass is only visible when the untrained arm is subjected to a disuse challenge is unknown, but suggests the existence of a protective mechanism that somehow potentiates the effects of unilateral training in a muscle wasting environment. Another hypothesis is that training the contra-lateral limb may inhibit the protein degradation pathways without activating the protein synthesis pathways; such effect would not be detected in steady conditions of basal protein degradation but would have visible consequences in conditions of severe muscle atrophy.

The main hypothesis that may explain the protective action of cross-education on skeletal muscle mass in the immobilized limb relies on the acute endocrine response triggered by exercise. Resistance exercise induces the secretion of anabolic and/or anti-catabolic circulating factors (Tremblay et al., 2004; Rubin et al., 2005; West et al., 2010; Rønnestad et al., 2011) and thereby enhances the anabolic environment. Similar principles have been proposed to explain blood-flow restriction-induced hypertrophy (Loenneke et al., 2012), where muscle occlusion might potentiate the release of stress responsive-anabolic factors during low-intensity resistance exercise. Anabolic factors released in the bloodstream in response to acute resistance exercise (Tremblay et al., 2004; Rubin et al., 2005; West et al., 2010; Rønnestad et al., 2011) include the androgenic steroidal hormone testosterone and the mitogenic hormones insulin growth factor 1 (IGF-1) and growth hormone (GH). Testosterone is a key regulator of skeletal muscle mass that directly promotes muscle protein synthesis (Urban et al., 1995) while also repressing the negative effect of the muscle specific atrophy related genes atrogin-1 and MuRF1 (Zhao et al., 2008). IGF-1 is the main activator of the Akt/mTOR pathway in muscle, while also carrying out signaling in other anabolic pathways including MAPK. Finally, GH is a direct activator of IGF-1 (Velloso, 2008) that may also enhance testosterone-induced protein synthesis (Vingren et al., 2010).

It is suggested that acute post-exercise hormone release may directly stimulate anabolism and prevent catabolism, leading to a net accretion in muscle proteins in both limbs. In the context of cross-education, a limitation to this hypothesis is that exercise-induced release of anabolic hormones in circulation is primarily associated with heavy resistance training protocols involving large muscles or group of muscles, while training smaller muscles or group of muscles do not elicit such response (Walker et al., 2004; West et al., 2010). Two studies have reported no improvement in muscle adaptation when the elbow flexors where trained alone or conjointly with leg and/or whole body exercises (Walker et al., 2004; West et al., 2010), refuting a potential systemic effect. However, one more recent study reported significantly improved strength training adaptations (1RM biceps curl and CSA of elbow flexor) when leg exercises were added to elbow flexors exercises only. In this study, elbow flexors were trained immediately after the legs at the peak of anabolic hormone release, supporting the theory that enhancing the anabolic milieu can result in muscle adaptation at the whole body level (Rønnestad et al., 2011).

The contribution of other potential stakeholders may also be considered. For example, little attention has been given to exercise-responsive myokines, including TNF-α and interleukines (IL) such as IL-6, IL-8, and IL-15 (Nielsen and Pedersen, 2007). These molecules may also play a role in hypertrophic adaptations, although the molecular mechanisms underlying their mode of action remain to be clearly defined. Reactive oxygen species (ROS), including nitric oxide (NO), may also contribute to elicit muscle protein synthesis and reduce protein degradation, notably by enhancing MAPK signaling (Kefaloyianni et al., 2006). Finally myostatin, a negative regulator of skeletal muscle mass, might play a role in chronic adaptation to unilateral training (Schiaffino et al., 2013). A 10-week high-intensity resistance training protocol involving only elbow flexors significantly decreased blood myostatin levels. The extent of this decrease was the same as following a 10-week high-intensity program engaging major muscle groups of the whole body (Walker et al., 2004), suggesting that both training regimes indifferently enhanced the systemic anabolic milieu. Altogether, current research suggests that the magnitude of changes driven by peripheral factors is probably fairly modest; however, even small increases in muscle hypertrophy are potentially relevant for some clinical populations.

## Limitations of the current studies

One important limitation of existing cross-educations studies is that despite an increase in strength, the vast majority of protocols did not induce an increase in muscle mass in the trained or untrained muscle or group of muscles (Bezerra et al., 2009; Farthing et al., 2011; Magnus et al., 2014; Kurobe et al., 2015; Beyer et al., 2016). Typically, the 3-week wrist training protocols used by Farthing (Farthing et al., 2009, 2011) and the 3-week elbow flexors training protocol used by Pearce (Pearce et al., 2013) were not sufficient to induce a hypertrophy of the trained muscles. Some studies also reported an increase in diameter in one, but not all trained muscles (Magnus et al., 2014; Kurobe et al., 2015). In this regard, a general limitation of cross-education studies is that often the applied training protocols do not reach the sufficient intensity, duration or overall load to induce an increase in muscle mass. With respect to traditional resistance exercise programs, there is a consensus suggesting that a concentric exercise at an intensity of minimum 60% of 1 repetition maximum (1RM) is necessary to induce muscle hypertrophy. Alternatively, low-intensity exercise (<50% 1RM) may have the same effect when completed to exhaustion (Schoenfeld, 2013). In terms of duration, recent studies have reported muscle hypertrophy following as few as 3 weeks of high intensity (>80% 1RM) training (Seynnes et al., 2007), with transient increases in muscle protein synthesis occurring after each exercise bout. However, to sustain muscle contraction, such training programs typically recruit a progressively increasing number of large motor units in the target muscle (McDonagh and Davies, 1984).

In contrast, the majority of recent cross-education studies have targeted muscles in the distal extremities such as the wrist flexors and extensors or intrinsic hand muscles (Farthing et al., 2009; Hortobágyi et al., 2011). This approach eliminates the potential confounding effects of strength gains in postural control musculature and typically provides a methodological advantage when examining the nervous system using techniques such as TMS and functional magnetic resonance imaging (fMRI). However, when considering applications of cross-education in injury rehabilitation, these methods lack functional relevance for translation into lower limb strength, mobility, and gait. Furthermore, training of small muscle groups is less likely to generate a significant systemic effect, which may provide additional benefits for exploiting the clinical applications of cross-education.

Early studies utilizing large or multiple muscle groups and longer duration training protocols are often quoted when discussing the absence of hypertrophy in the untrained limb (Narici et al., 1989; Housh et al., 1992). While it is true that no significant increase in CSA of the untrained muscle were reported, it should also be acknowledged that there was also no significant increase in force output of the untrained limb (Narici et al., 1989; Housh et al., 1992). Further, Housh et al. (1992) stated that a trend for hypertrophy was observed, with as much as 14% increase in CSA for the untrained rectus femoris. Another early study that found no change in CSA of the untrained muscle reported significant but modest increases for the trained limb (5%), and used a protocol designed specifically to increase strength without inducing hypertrophy (by performing only concentric movements, and 2 days per week training frequency; Ploutz et al., 1994). One more recent study using a 6-week duration protocol used ultrasound to assess muscle thickness, reporting a small magnitude on increase (4.1%) in the trained limb only (Farthing et al., 2005). Given that the expected magnitude of transfer of force is approximately half that observed in the trained limb, it may be difficult to detect equivalent transfer of hypertrophy in the untrained limb, even with sensitive measuring techniques such as MRI and pQCT.

The results from another early study (Brown et al., 1990) indicate that there may be a need to investigate the inner structure of the muscle (i.e., the muscle fiber CSA) rather than the thickness or diameter of the whole muscle. In addition, a variety of methods displaying different levels of reproducibility and variability have been used across studies, suggesting that caution needs to be taken when making comparisons between studies (see Table 1). At the whole muscle level, an increase in type IIb fiber cross-sectional area (the fast-glycolytic fibers that are adapted for resistance exercise, using glucose as their main substrate) may not significantly contribute to an increase in diameter of the whole muscle, while still allowing more force to be produced. Another factor that warrants investigation is the functional protein synthesis response of the muscle. In healthy adult muscle, an increase in muscle mass occurs as a result of an increase in muscle protein synthesis (hypertrophy) rather than the formation of new muscle fibers. Assessing muscle protein synthesis *in vivo*, typically using radiolabeled tracer infusion (Lamon et al., 2016), provides a more accurate and specific picture of the muscle functional response than measuring muscle diameter does. One of the limitations of a even gold standard cross-sectional scans (such as MRI and pQCT) that are used to assess muscle thickness is that they are unable to distinguish between muscle tissue and intramuscular fluid. As a consequence, increases in muscle diameter might not only reflect an accumulation of contractile proteins, but also training-induced inflammatory responses (muscle oedema). In contrast, measuring protein synthesis *in vivo* allows to capture a temporal snapshot of the muscle functional response to exercise. Repeated increases in muscle protein synthesis in response to each exercise session may occur in the untrained limb as a result of cross-education, even if not significantly contributing to muscle hypertrophy overall (Phillips, 2000).

**Table 1:** **Studies reporting a change in muscle adaption with unilateral training**.

**References**	**Muscle thickness assessment**	**Muscle**	**Intervention in the non-training limb**	**Effect of training on contralateral muscle size**	**Effect of no training on contralateral muscle size**	**Variability**
Brown et al., 1990	Computerized tomography scans	Elbow flexors (biceps brachii and brachialis)	N/A	No change	N/A	N/A
Bezerra et al., 2009	MRI	Quadriceps	N/A	No change	No change	ICC = 0.99
Farthing et al., 2009	B-Mode ultrasound (SSD-500; Aloka)	Posterior medial forearm (flexor carpi ulnaris and flexor digitorum superficialis)	Immobilization	−1.1% (NS)	−4.3% (^*^)	CV = 1.4–1.5%
Magnus et al., 2010	B-Mode ultrasound (SSD-500; Aloka)	Biceps brachii	Immobilization	2.2%	−2.8% (^*^)	CV = 1.4–1.5%
Magnus et al., 2010	B-Mode ultrasound (SSD-500; Aloka)	Triceps brachii	Immobilization	3.4%	−5.2% (^*^)	CV = 1.4–1.5%
Farthing et al., 2011	B-Mode ultrasound (SSD-500; Aloka)	Posterior medial forearm (flexor carpi ulnaris and flexor digitorum superficialis)	Immobilization	−4.72% (NS)	−1.66% (NS)	CV = 1.4–1.5%
Pearce et al., 2013	Nemio20 premium compact ultrasound (Toshiba)	Elbow flexors (biceps brachii and brachialis)	Immobilization	0% (NS)	−6% (^*^)	CV = 2.4%
Magnus et al., 2014	Portable ultrasound scanner (LOGIQ e BTO8, GE Healthcare)	Supraspinatus	N/A	−0.5% (NS)	+0.5% (NS)	CV = 3.7%
Magnus et al., 2014	Portable ultrasound scanner (LOGIQ e BTO8, GE Healthcare)	Anterior deltoid	N/A	+5.1% (NS)	−7.35% (NS)	CV = 3.7%

*ICC, intraclass correlation coefficient; CV, coefficient of variation; NS, non-significant when compared to baseline*;

* significantly different from baseline, p < 0.05

## Conclusion

The cross-education effect is mainly explained by increased neural drive originating from the untrained motor cortex. However, understanding the positive effect of cross-education on skeletal muscle adaptation requires the consideration of factors beyond the brain. Achieving a greater understanding of the physiological mechanisms contributing to cross-education is important to more effectively explore its effects and potential applications in rehabilitation of unilateral movement disorders or injury.

## Author contributions

AH and SL equally contributed to the design, the redaction and the edition of this article.

## Funding

AH and SL are supported by the Deakin University Central Research Scheme. SL is supported by a Discovery Early Career Research Award (DECRA) from the Australian Research Council (ARC) (DE150100538).

### Conflict of interest statement

The authors declare that the research was conducted in the absence of any commercial or financial relationships that could be construed as a potential conflict of interest.
